# Structural Investigations of Interactions between the Influenza a Virus NS1 and Host Cellular Proteins

**DOI:** 10.3390/v15102063

**Published:** 2023-10-07

**Authors:** Morgan E. Blake, Alex B. Kleinpeter, Alexander S. Jureka, Chad M. Petit

**Affiliations:** Department of Biochemistry and Molecular Genetics, University of Alabama at Birmingham, Birmingham, AL 35294, USA; moreliz@uab.edu (M.E.B.);

**Keywords:** influenza, NS1, non-structural protein 1, structure, structural virology

## Abstract

The Influenza A virus is a continuous threat to public health that causes yearly epidemics with the ever-present threat of the virus becoming the next pandemic. Due to increasing levels of resistance, several of our previously used antivirals have been rendered useless. There is a strong need for new antivirals that are less likely to be susceptible to mutations. One strategy to achieve this goal is structure-based drug development. By understanding the minute details of protein structure, we can develop antivirals that target the most conserved, crucial regions to yield the highest chances of long-lasting success. One promising IAV target is the virulence protein non-structural protein 1 (NS1). NS1 contributes to pathogenicity through interactions with numerous host proteins, and many of the resulting complexes have been shown to be crucial for virulence. In this review, we cover the NS1-host protein complexes that have been structurally characterized to date. By bringing these structures together in one place, we aim to highlight the strength of this field for drug discovery along with the gaps that remain to be filled.

## 1. Introduction

The Influenza A virus is a significant public health concern that causes recurrent, seasonal epidemics in human populations with occasional global pandemics resulting in substantial levels of morbidity and mortality. These seasonal epidemics result in over 200,000 hospitalizations [1] and approximately 35,000 deaths [2] in the United States alone. The ability of influenza A viruses to adapt to various hosts and to undergo genetic reassortment (i.e., mixing of gene segments from unique viruses), ensures constant generation of unique strains. Each of these strains have varying degrees of pathogenicity, transmissibility, and pandemic potential. The most recent reassortment occurred in 2009 when a novel influenza virus reassortant circulating in pigs began infecting humans resulting in a pandemic strain commonly known as “swine flu”. This newly emergent 2009 H1N1 pandemic strain rendered vaccines ineffective [3], however treatment with the antiviral Oseltamivir (Tamiflu) was associated with a decrease in morbidity and mortality in infected patients [4]. This demonstrates the importance of both prevention (vaccines) and treatment (antivirals) as strategies to combat influenza viruses in the future. Moving forward, this will require a comprehensive understanding of the molecular determinants of pathogenesis and replication of the virus.

Influenza A virus (IAV) is a negative-sense, single-stranded RNA virus in the family *Orthomyxoviridae*. IAV’s genome consists of eight segments (PB1, PB2, PA, HA, NA, NP, M, NS) that were initially assumed to encode ten proteins. The Polymerase Basic 1 (PB1), Polymerase Basic 2 (PB2), and Polymerase Acidic (PA) proteins comprise the tri-partite RNA-dependent RNA polymerase (RdRp) complex responsible for transcription and replication of the IAV genome [5]. Hemagglutinin (HA) and neuraminidase (NA) are the IAV surface proteins involved in virus entry (HA) and release (NA) [6,7,8,9]. Antigenic differences in HA and NA are used to classify influenza viruses into distinct subtypes (i.e., H1N1, H7N9, H5N1, etc.) [10]. To date, there are 16 distinct HA subtypes and 9 distinct NA subtypes [11]. If you include the influenza-like viruses found in bats that are phylogenetically close to IAVs but cannot reassort with them [12,13], the total number of HA and NA subtypes increases to 18 and 11, respectively. The nucleoprotein (NP) is a single-stranded RNA binding protein that encapsidates the viral genome to form the ribonucleoprotein (RNP) particle [14]. The M and NS gene segments generate viral mRNAs that undergo alternative splicing, resulting in each segment typically encoding two distinct proteins [15]. The M gene segment encodes the matrix 1 (M1) and matrix 2 (M2) proteins, both of which are structural. The M1 protein is the most abundant protein within the virion particle and participates in nuclear RNA export, virion particle assembly, and virus disassembly [16]. M2 proteins are homotetrameric, type III integral membrane proteins that function as proton channels and are essential to influenza replication [17,18]. The NS gene segment encodes non-structural protein 1 (NS1) and the nuclear export protein (NEP). NS1 is known to play critical roles during viral infection, such as suppressing the innate immune response and acting as a virulence-modulator [19,20]. NEP was initially thought to have no structural function within the virion, resulting in its original designation as non-structural protein 2 (NS2). However, it was later renamed to NEP after small quantities of the protein were found to be present in the virion where it interacted with M1 [21,22,23]. NEP is a multifunctional protein implicated in the export of vRNP complexes from the host nucleus [24,25,26] as well as regulation of the RNA-dependent RNA polymerase (RdRp), which has been linked to the zoonosis of H5N1 influenza strains [27,28,29].

Interestingly, additional accessory proteins have been found to be expressed using a variety of mechanisms, such as alternative splicing and ribosomal frameshift. These proteins include PA-X [30], PA-N155 [31], PA-N182 [31], PB1-F2 [32], PB1-N40 [33], PB2-S1 [34], M42 [35], and NS3 [36], which brings the total number of potential proteins expressed by the influenza genome up to eighteen. This greatly expands the number of proteins potentially expressed by influenza that are not directly involved in the replication process. The most well-characterized protein not directly involved in IAV replication is NS1. Only 10–15% of NS1 mRNA is spliced to generate NEP [37,38], resulting in significant accumulation of NS1 during infection. In the absence of NS1, viral replication is significantly attenuated due to the rapid and uncontrolled induction of the cellular type I interferon response (IFN-α/β) in response to the infection [39]. NS1 accomplishes its functions by interacting with cellular proteins in both the nucleus (e.g., CPSF30 [40,41,42]) and the cytoplasm (e.g., RIG-I [43,44,45,46], PKR [47,48], PI3K [49,50,51,52,53]). Because these interactions occur in different subcellular compartments, the regulation of NS1 translocation is critical to NS1 function [20]. Most of these interactions contribute to the central role of NS1 during IAV infection: the antagonism of the type-I interferon response [19,20,54,55,56]. NS1’s potent antagonism of the host innate immune response to infection means that it can play a key role in IAV replication [39,57,58], pathogenicity [58,59,60,61,62], and host range [36,63,64,65]. Because of this critical role in the viral lifecycle, numerous studies have highlighted NS1 as a high-value target for the development of anti-influenza compounds [66,67,68,69,70,71,72,73].

Despite NS1 being a high-valued target for the development of antivirals, there are currently no antiviral treatments that target NS1 at any stage of clinical trial [74,75]. We posit that the lag in development of antivirals targeting NS1 would be alleviated if the structural aspects of each interaction and the functional consequences of abrogating them on viral replication and pathogenesis were more understood. There have been numerous interactome studies focusing on identifying interactions between NS1 and host cell proteins [76,77,78,79]. Validating each of these many interactions is a large undertaking, making progress on this front slower than desired. The purpose of this review is to provide a brief summary of each interaction that has been verified as interacting directly with NS1 and provide structural analysis when available.

## 2. NS1 Structure

**Figure 1:**
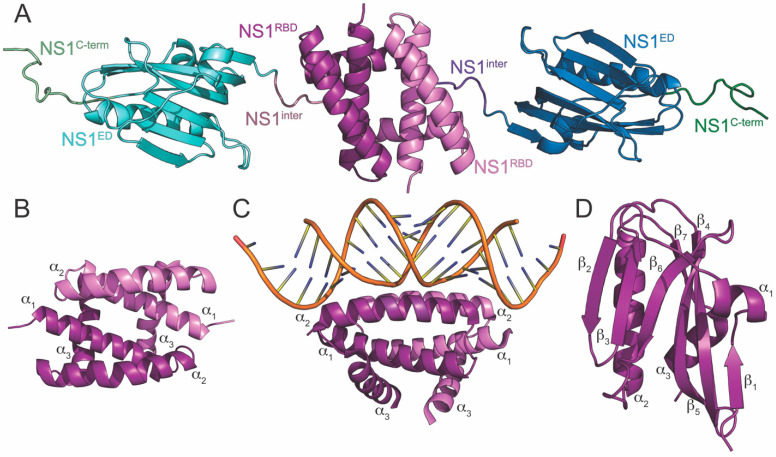
Crystal structures of NS1 and its domains. (**A**) Full-length NS1 derived from the A/Vietnam/1203/2004 (H5N1) strain of influenza. Two mutations (R38A and K41A) were needed to make the full-length protein amenable for crystallographic studies. The four discrete structural regions of NS1 are labeled as NS1^RBD^, NS1^inter^, NS1^ED^, and NS1^C-term^ and color coded in violet, blue violet, blue, and green, respectively. Each monomer of full-length NS1 is color coded for each region using different shades of the same color. (**B**) The NS1^RBD^ derived from the A/Brevig Mission/1/1918 (H1N1) strain of influenza. (**C**) The NS1^RBD^ derived from the A/Puerto Rico/8/1934 (H1N1) strain of influenza bound to dsRNA. (**D**) The NS1^ED^ derived from the A/Brevig Mission/1/1918 (H1N1) strain of influenza. The secondary structural elements of each independently folding domain of NS1 are labeled.

The NS1 protein of influenza A is a highly multifunctional protein that ranges in sequence length from 215 to 237 amino acids (~26kDa) depending on the strain from which it is derived [80,81]. NS1 is divided into four discrete structural regions (Figure 1A). Its N-terminal RNA binding domain (NS1^RBD^) is composed of amino acid residues 1-73 and is primarily responsible for NS1’s RNA binding activity [82,83,84,85]. The effector domain (NS1^ED^), consisting of amino acid residues 86-205, interacts with numerous host factors involved in the innate immune response to infection. Between the NS1^RBD^ and NS1^ED^ is a short, flexible linker (~residues 74-85). The functional importance for this linker region is poorly understood; however, a naturally occurring deletion of residues 80-84 has been observed in some highly pathogenic H5N1 strains of influenza [86]. Finally, NS1 contains an unstructured C-terminal tail (~residues 206-237) of varying length depending on strain (NS1^C-term^). Numerous host interactions have been reported to be mediated by the NS1 C-terminus [87,88,89], some of which affect pathogenicity [90,91]. The three-dimensional structures of individually expressed NS1^RBD^ and NS1^ED^ have been extensively studied [92,93,94,95,96,97,98,99,100,101,102,103]. However, little is known regarding the interdomain contacts and quaternary structure of NS1. This is mainly due to the propensity of full-length NS1 to aggregate at relatively low concentrations in solution [103]. Indeed, the few published full-length NS1 structures include mutations to mitigate its aggregation, allowing for its amenability to traditional structural techniques [104,105].

## 3. NS1^RBD^

The three-dimensional structure of the NS1^RBD^ has been solved using X-ray crystallography [98] and nuclear magnetic resonance (NMR) [82,95]. It is a symmetric homodimer (Figure 1B) consisting of six α-helices (three from each monomeric unit), two of which (α2 and α2′) form a “track” that interacts with the major groove of dsRNAs [106,107]. Basic residues along this RNA-binding track are heavily involved in the interaction, as can been seen in the high-resolution structure of the NS1^RBD^ bound to dsRNA (Figure 1C) [106,108]. Specifically, Arg-38 and Lys-41 are required for this interaction, and recombinant IAVs harboring mutations at these positions are severely attenuated [83]. NS1^RBD^ sequence and structure is very well-conserved among different strains of IAV [82,95,99,106]; however, subtle differences between strains have recently been identified. For example, a recent study showed that a potential salt bridge present in the NS1^RBD^ of the A/Brevig Mission/1/1918 (H1N1) strain of IAV (1918^H1N1^) facilitates a strain-dependent interaction with the second caspase activation and recruitment domain (CARD2) of the retinoic acid inducible gene-I (RIG-I) [95,109].

## 4. Interdomain Linker

The interdomain linker is a short, flexible linker (~residues 74-85) that connects the two independently folding domains of NS1 (NS1^RBD^ and NS1^ED^) [105]. Although the amino acid sequence of the linker is conserved among influenza A viruses, its impact on overall NS1 function is not known [110]. There have been several studies that have begun to characterize its role in NS1 function and in the context of influenza infection. For example, it has been shown that a naturally occurring deletion in the interdomain linker (residues 80-84) increases viral replication and pathogenicity [86]. This deletion is found in some highly pathogenic H5N1 influenza strains [86]. It has also been shown that mutating the interdomain linker of NS1 inhibited its ability to suppress the innate immune response, decreased overall viral replication, and abrogated NS1’s ability to activate the PI3K/Akt anti-apoptotic pathway [110]. Finally, mutations in the interdomain linker of NS1 were identified as possible enhancers of zoonotic transmission [111,112]. Follow-up studies are needed to fully characterize the role that the interdomain linker plays in the regulation of NS1 function, influenza pathogenesis, and zoonotic transmission.

## 5. NS1^ED^

The NS1^ED^ is significantly less well-conserved across strains of IAV than the NS1^RBD^. This is likely due to the numerous strain-dependent host interactions that it participates in during IAV infection [76]. Like the NS1^RBD^, structures of the NS1^ED^ have been solved using both X-ray crystallography [92,94,96,97,100,101,102] and NMR [103]. The structure of the monomeric unit of the NS1^ED^ consists of three α-helices and seven β-strands (Figure 1D). The core of the protein is arranged as a long central α-helix surrounded by a twisted antiparallel β-sheet. The independently expressed NS1^ED^ dimerizes rather weakly compared to the NS1^RBD^, and the importance of its dimerization is still unclear [103,113].

## 6. NS1^C-term^

**Figure 2:**
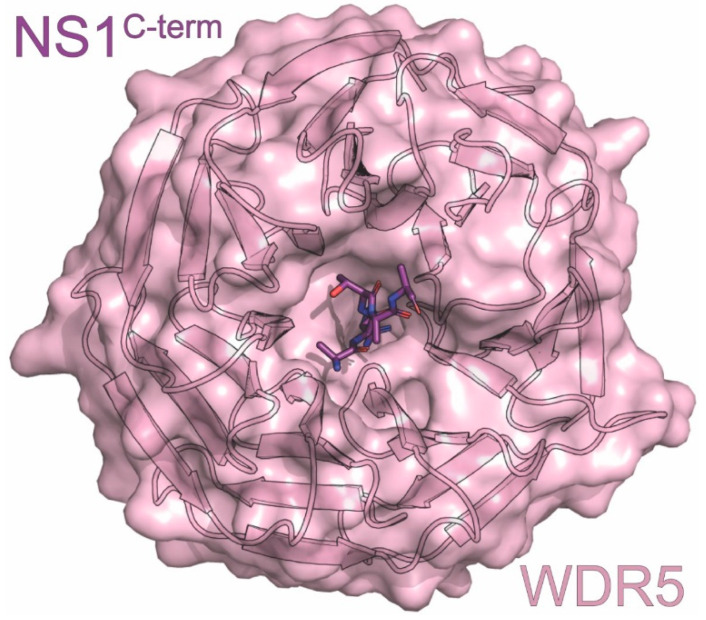
Crystal structure of the WDR5 in complex with NS1. The structure consists of WDR5 (pink) in complex with the carboxyl terminus of NS1 (purple) derived from the A/Udorn/307/1972 (H3N2) strain of influenza. The PDB ID for this structure is 4O45.

Although many constructs used for solving NS1^ED^ structures have included residues from NS1’s C-terminal tail (~residues 206-230), no electron density for this region has been observed [92,96,114]. It is possible, however, that this region assumes some structure upon interaction with host binding partners. Studies utilizing short peptides derived from NS1’s C-terminus have allowed the identification of binding partners such as the chromatin-associated factor WDR5 [115] (Figure 2) [116]. Further studies are necessary to determine the contribution of this region to overall NS1 structure and function.

## 7. CHD1

CHD1 is a chromatin remodeling protein that can be found in the nuclei of host cells [117,118,119]. It consists of two chromodomains, an SWI/SNF domain for its chromatin remodeling functions and a C-terminal DNA binding domain [115,120]. CHD1 recruits post-transcriptional initiation and splicing factors to the DNA through its recognition of trimethylated histone H3 lysine 4 (H3K4), a sign of actively transcribed chromatin [115,121]. Although many histone binding proteins cradle the free N-terminal amine of histone H3 in a deep, negatively charged pocket, CHD1 has a shallow open pocket [115]. This shallow binding pocket allows the C-terminus of NS1 to imperfectly mimic the histone tail and bind to CHD1. Unlike most other proteins that bind histones, however, its interaction with NS1 specifically affects CHD1’s binding pool of actively transcribed DNA.

The C-terminal tail of NS1 is an unstructured region with binding sites for many different types of host proteins [90,94,122]. NS1 introduces an additional binding site to this region through a sequence that mimics the histone H3 tail. The “^226^ARSK^229^” sequence is chemically analogous to histone H3’s N-terminal “^1^ARTK^4^”, even retaining its ability to be post-translationally modified by lysine methyltransferases Set1 and Set7/9 or acetyltransferase TIP60 [87]. One key difference is that the methylation that would mark a histone as transcriptionally active is unable to be removed from NS1 by demethylase LSD1, allowing it to continuously be recognized by CHD1 [115].

**Figure 3:**
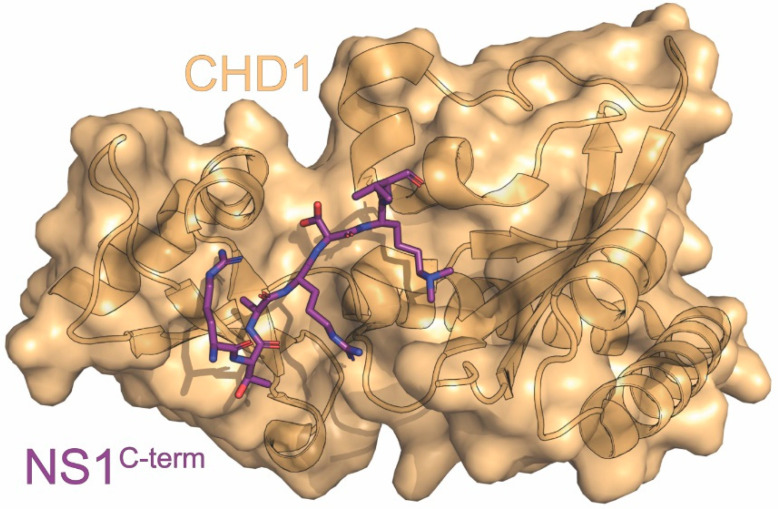
Crystal structure of CHD1 in complex with NS1^C-term^. The structure consists of CHD1 (orange) in complex with the carboxyl terminus of NS1 (purple) derived from the A/Hong Kong/5/1983 (H3N2) strain of influenza. The PDB ID for this structure is 4O42.

CHD1 was found to bind to the di- or tri-methylated NS1 C-terminal tail through immunoprecipitation and western blot with purified NS1 peptide, ITC, and an X-ray crystal structure of CHD1’s chromodomains complexed with NS1 C-terminal tail peptide (Figure 3) [87,115]. The two chromodomains bind to the NS1 “^226^ARSK^229^” sequence through two types of interactions: 1) cation-π interactions between the methylated K229 of NS1 and W322 and W325 from CHD1 chromodomain 1, reinforced by E272 from chromodomain 1 interacting with the lysine through its negative charge and 2) a series of hydrogen bonds either directly or through ordered solvent [115]. We note that dimethylated NS1 was preferred to trimethylated, due to the third methyl being solvent exposed [120]. The crystal structure obtained was almost identical to chromodomain 1 complexed with trimethylated histone H3, with the main difference being A226 of NS1’s 60º rotation relative to A1 of histone H3. This rotation allows an additional salt bridge between R224 of NS1 and D408 and D425 of CHD1 [115].

NS1’s C-terminal tail contains an imperfect histone mimic due to the lack of a free N-terminal amine that is normally required for interactions with histone binding factors, but this serves as an advantage for IAV. Its ability to bind to CHD1’s shallow binding pocket allows NS1 to target its effects to actively transcribed chromatin, aiding its efforts to curtail virus-induced production of antiviral genes. Additionally, it is unable to be demethylated, allowing it to escape from normal host regulation of this process. The mechanistic underpinnings of NS1’s hijacking of CHD1 still need to be elucidated, but this interaction adds another host to NS1’s toolkit for downregulation of host genes that could be a novel target for antiviral drugs. 

## 8. CrkII

The Crk family is a set of important adaptor proteins that are required for many essential cellular signaling processes. They contain Src-homology domains (SH) that bind to proline-rich regions on their ligands. CrkL and CrkII have one SH2 and two SH3 domains, while CrkI, an alternative splicing product of CrkII, has one of each [123,124]. The SH2 and SH3 domains have binding pools that largely overlap with a few exceptions, and the Crk proteins exert their function by bringing together proteins that interact with these domains [125,126]. A subset of IAV strains is able to prevent these linkages through host–pathogen interactions involving their NS1 proteins and the Crk family of proteins. For these strains, the NS1 C-terminal tail encodes a proline-rich motif (PRM) that can bind to the Crk family of proteins. The PPLPPK motif at residues 212–217 of NS1 is a perfect class II SH3-binding motif that is highly conserved in avian strains but is only present in a handful of human strains, notably including the 1918^H1N1^ pandemic strain [125,127]. P212, P215, and K217 were found to be especially important, with a T215P mutation proving sufficient to confer SH3 binding to the normally binding-incompetent WSN strain [126,127]. Most PRMs bind to SH3 domains with low specificity, but NS1 binds to CrkII’s SH3 in a highly specific manner, leading Shen et al. to investigate the structural mechanisms behind the interaction [116,127].

**Figure 4:**
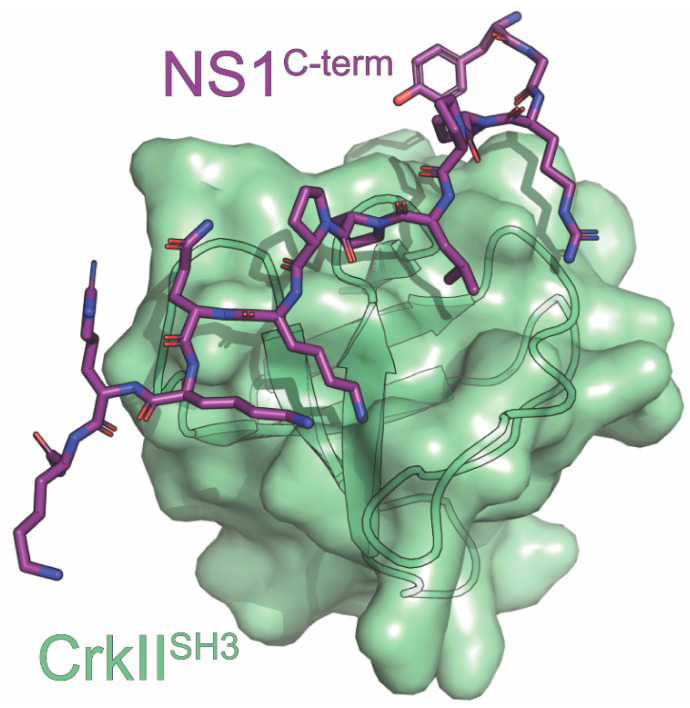
Crystal structure of CrkII^SH3^ in complex with NS1. The structure consists of CrkII^SH3^ (light green) in complex with the carboxyl terminus of NS1 (purple) derived from the A/Brevig Mission /01/1918 (H1N1) strain of influenza. The PDB ID for this structure is 5UL6.

Shen et al. structurally defined the interaction of the PRM of NS1 derived from the 1918^H1N1^ strain of influenza (1918 NS1^PRM^) and the N-terminal SH3 domain of CrkII (nSH3^CrkII^) [116]. The methods used in this study included X-ray crystallography, NMR relaxation dispersion, and fluorescence spectroscopy (Figure 4) [116]. Using these methods, they revealed a bipartite binding interface that was responsible for NS1’s increased selectivity. The N-terminal region of the 1918 NS1^PRM^ interacts with CrkII’s SH3 domain via hydrophobic interactions mediated by the PPLP motif. This observation is consistent with other SH3-PRM complexes. The C-terminal region of the 1918 NS1^PRM^ forms two sets of electrostatic interactions with a negatively charged surface of the nSH3^CrkII^ domain. A salt-bridge network forms between the ε-amino group of K217 of the 1918 NS1^PRM^ and D147, E149, and D150 of nSH3^CrkII^. This network has been observed in CrkII complexes with Abl kinase and C3G, but it is not common. Additionally, a bidentate hydrogen bond forms between E166 of nSH3^CrkII^ and the backbone amides of Q218 and K219 of the 1918 NS1^PRM^. No other SH3 domains besides nSH3^CrkII^ have been observed to form both sets of interactions with its binding partner, which would explain 1918 NS1^PRM^’s high specificity for CrkII.

Shen et al. also compared the binding kinetics between nSH3^CrkII^ and the 1918 NS1^PRM^ to a previously identified nSH3^CrkII^ interaction partner, the PRM encoded by c-Jun-N-terminal kinase 1 (JNK^PRM^) [116]. The *K*_d_ of the 1918 NS1^PRM^-nSH3^CrkII^ complex was measured to be 6nM, which is 3300-fold lower than the *K*_d_ measured for the JNK^PRM^- nSH3^CrkII^ complex. For reference, the *K*_d_ values for SH3-PRM complex typically range between 1 and 10µM [128]. Additionally, the *k*_on_ for NS1 binding to nSH3^CrkII^ is 100-fold faster, while the *k*_off_ for the 1918 NS1^PRM^-nSH3^CrkII^ complex is 35-fold slower compared to the *k*_on_ and *k*_off_ for the JNK^PRM^- nSH3^CrkII^ complex. Taken together, the significantly increased *k*_on_, decreased *k*_off_, and decreased *K*_d_ observed for the 1918 NS1^PRM^-nSH3^CrkII^ complex offers a clear mechanistic explanation for NS1’s ability to competitively inhibit the interaction between JNK^PRM^ and nSH3^CrkII^.

Since the Crk family binds to numerous proteins that facilitate various processes in the host cell, NS1’s displacement of these proteins influences several cellular processes. For example, NS1 suppresses the activation of the host antiviral immune response by outcompeting the interactions between the Crk family and kinases such as JNK1 and Abl [129,130,131]. The interaction between NS1 and JNK1 is also thought to suppress apoptosis that is normally triggered upon viral infection. However, it has been suggested that the CrkII modulation of the apoptotic pathway may not be as important as its counterparts in this process [125,129]. Furthermore, Ylosmaki et al. showed that NS1 binding to Crk proteins can translocate them into the nucleus, leading to a change in nuclear protein phosphorylation. This could also lead to changes in apoptosis since CrkII has been reported to bind to nuclear cell cycle regulator Wee1 along with activating caspases [126,132]. Finally, Crk binding is involved in PI3K signaling. During infection, NS1 hyperactivates PI3K signaling by binding to both p85β of PI3K and the Crk family. The interaction between NS1 and PI3K is further discussed later in this review [127,133]. In summary, the heightened selectivity of interactions between NS1 and the Crk family of proteins, especially CrkII, facilitates control of many important cellular processes by the IAV that prove beneficial for viral fitness.

## 9. Scribble

Proper cell polarity is essential for tissue development, organization, and function. It ensures that macromolecules, including cellular junctions, are located in their proper positions. This process is controlled by the actions of three competing protein modules: Crumbs, PAR, and Scribble [134]. The Scribble module consists of the leucine-rich repeats and the PDZ domain (LAP) protein Scribble bound to Dlg and Lgl, and the three are normally located together in the cytoplasm near the plasma membrane [135,136,137]. While previously thought to only be scaffolding proteins, PDZ proteins have since been shown to have more involvement in cellular processes, such as Scribble’s proapoptotic functions [138,139]. Scribble enacts its effects via interactions with different proteins through its various domains, including its four PDZ domains, which multiple viruses exploit [136,140].

**Figure 5:**
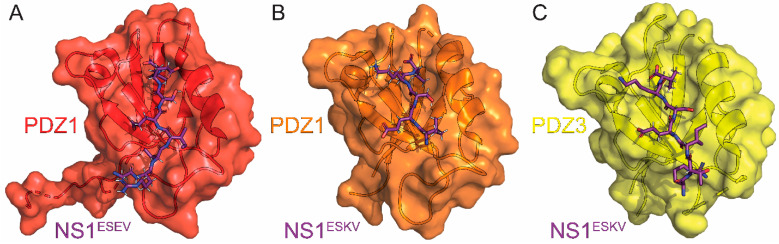
Crystal structures of Scribble in complex with NS1. (**A**) The first PDZ domain (PDZ1) of scribble (red) in complex with the PDZ binding motif (PBM) of NS1 derived from the A/Vietnam/1194/2004 (H5N1) (purple). (**B**) PDZ1 (orange) in complex with the PBM of NS1 derived from the A/Chicken/Hubei/327/2004 (H5N1) strain of influenza (purple). (**C**) The third PDZ domain (PDZ3) of scribble (yellow) in complex with the PBM of NS1 derived from the A/Chicken/ Hubei/327/2004 (H5N1) strain of influenza (purple). The PBM of NS1 proteins derived from the A/Vietnam/1194/2004 (H5N1) consist of ESEV residues at their carboxyl terminus (NS1^ESEV^), while the PBM of NS1 proteins derived from the A/Chicken/Hubei/327/2004 (H5N1) strain of influenza consist of ESKV residues at their carboxyl terminus (NS1^ESKV^). The PDB IDs for these structures are 7QTO, 7QTP, and 7QTU for PDZ1:NS1^ESEV^, PDZ1:NS1^ESKV^, and PDZ3:NS1^ESKV^, respectively.

There are several NS1 proteins that encode a PDZ-binding motif (PBM) at their extreme C-termini. We note that PBMs are not encoded universally by all NS1 proteins. Of the NS1 proteins that do, there are differences in their sequences when comparing avian and human strains [141]. About 90% of avian isolates have ESEV or ESKV at the C-terminus, while human strains usually have an RSKV. These follow Scribble’s class I PDZ recognition sequence motif of X-T/S-X-V/L_COOH_. However, due to their varying sequences, they have different effects on pathogenicity and virulence. Using peptides that encode these NS1 PBMs, the interaction between the PBM of NS1 and Scribble has been studied via pulldowns, co-IP, in vitro binding assays with recombinant purified protein, ITC, and X-ray crystallography [88,89,137,142,143]. The avian sequences ESEV and ESKV have been found to interact with Scribble’s PDZ domains, while the human isolate sequences lack this ability [88,89,137,142,143]. To confirm that the PBMs were primarily responsible for these interactions, chimeras were made in which the human and avian PBMs were interchanged between the two NS1 proteins. This interchange of PBMs lead to reversal of the previously observed interactions between the human and avian strains with Scribble. However, it was shown that avian NS1 with a mutated EAEA non-functional PBM was still able to bind more tightly than human NS1, suggesting that other regions could also be involved [89,137]. It is still unclear which PDZ domain of Scribble interacts with the PBM encoded by NS1. Specifically, in vitro binding assays showed that full-length NS1 was unable to bind to individual PDZ domains of Scribble. However, it was determined that a tandem construct encoding PDZ1 and PDZ2 was necessary and sufficient for the interaction. Finally, all four individual PDZ domains of scribble were able to bind 10-mer peptides encoding the extreme NS1^C-term^, and these complexes were used to solve the high-resolution crystal structures (Figure 5) [137,143].

The ITC and X-ray crystallography experiments performed by Javorsky et al. revealed several important interactions that were also confirmed via mutational analysis [143]. The PBM of NS1 was found in the canonical ligand-binding pocket of the PDZ domain, and the structures overlaid with Scribble complexed with an endogenous ligand generated RMSD values ranging from 0.57 to 0.75Å. Their structures of PDZ1 complexed with either ESEV or ESKV (Figure 5A,B) and PDZ3 (Figure 5C) with ESKV identified a number of hydrogen bonds and van der Waals contacts that can be disrupted to abolish binding. The conserved Histidine found on the α2 helix of each PDZ domain is critical to the interaction, as mutating it to an alanine abolished binding in each case except for PDZ2, which enhanced binding. Both PBMs encoding ESEV and ESKV interacted with all four PDZ domains with affinities in the low micromolar. The relative binding affinities for the PBM encoding ESEV to each PDZ domain were PDZ4 > PDZ3 > PDZ2 > PDZ1. For the PBM encoding ESKV, the relative affinities were PDZ2 > PDZ3 >PDZ1 with no interaction detected for PDZ4. Compared to the endogenous ligand β-PIX, the NS1 PBM had lower affinity for PDZ1, higher for PDZ2, and higher for PDZ4 since β-PIX is also unable to bind this domain. This provides support for a mechanism by which NS1 outcompetes Scribble’s endogenous ligands to prevent its functions.

Scribble function has been shown to be disrupted by NS1 proteins derived from avian IAVs by blocking its interaction with natural ligands as well as causing mislocalization of Scribble. Specifically, Scribble is typically found in the cytoplasm adjacent to the plasma membrane, but it is found in perinuclear puncta with NS1 and Dlg1 during infection with avian IAVs [88,137]. This mislocalization results in disruption of tight junctions that allows for increased viral dissemination within the host and decreased apoptosis that is crucial for early survival of the virus [88,137]. However, the effect on NS1’s interferon antagonism is unclear in this context. One study showed that avian NS1’s reduction in interferon-induced STAT phosphorylation was hindered by a non-functional PBM, while another showed that mutants and wild-type PBMs had no difference in their blocking of IFNβ induction [89,142]. Another aspect that is not fully understood is the PBM’s synergistic upregulation of Akt phosphorylation with the PI3K binding loop [142]. What is clear, however, is that disruption of the normal function of Scribble by NS1 is able to regulate many different aspects of cellular function to benefit viral replication.

## 10. MORC3

Microrchidia 3 (MORC3, also known as NXP2, ZCW5, ZCWCC3, or KIAA0136) is a human ATPase involved in the host antiviral response, transcription regulation, and chromatin remodeling [144]. Pertinent to this review, MORC3 participates in the antiviral response through its recruitment to promyelocytic leukemia nuclear bodies (PML-NBs). It consists of a GHKL (gyrase, Hsp90, histidine kinase, and MutL)-type ATPase domain, a CW-type zinc finger domain, and a coiled-coil region [145]. Upon localization to PML-NBs, it recruits and activates p53 to induce cellular senescence among other antiviral defenses [146,147]. In its native state, MORC3 function is inhibited by its own CW domain (MORC3-CW) that self-associates with its neighboring ATPase domain to prevent DNA binding, which is required for catalysis. MORC3 becomes activated when its CW domain binds to the tail of histone H3, preferably histone H3 proteins that are tri-methylated at the 4th lysine residue (H3K4me) [148]. Many viruses have developed ways to overcome this regulation through histone mimicry. One such histone mimic includes the C-terminal tail of NS1 derived from a number of IAV strains [87].

**Figure 6:**
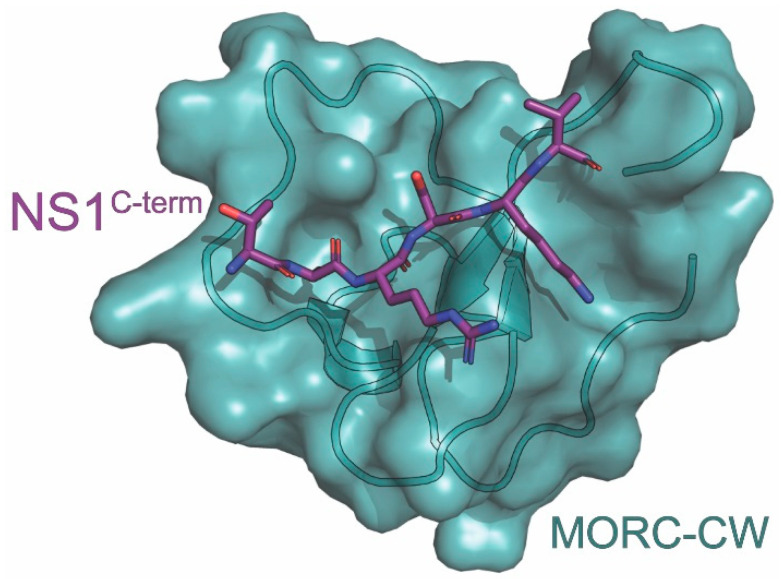
Crystal structure of MORC3-CW in complex with NS1. The structure consists of MORC3-CW (teal) in complex with the carboxyl terminus of NS1 (purple) derived from the A/Wyoming/03/2003 (H3N2) strain of influenza. The PDB ID for this structure is 6O5W.

The interaction between the NS1^C-term^ and MORC3-CW has been characterized by Zhang et al. using NMR spectroscopy and X-ray crystallography (Figure 6) [149]. NMR chemical shift perturbation analysis revealed direct binding between the MORC3-CW and residues 222-230 of the NS1^C-term^. It was also found that using an NS1^C-term^ peptide trimethylated at K229 (NS1^K229me3^) increased its binding affinity with MORC3-CW. To solve the X-ray crystal structure of the complex, the NS1^C-term^ (residues 225-230) was fused to MORC3 (residues 407-455) using a GGSG linker. NMR analysis using heteronuclear single quantum coherence (HSQC) spectra determined that the fusion protein consisted of separated domains. The X-ray crystal structure of the MORC3-CW:NS1^C-term^ complex was then solved at 1.41 Å resolution. The structure revealed that the MORC3-CW folds into a compact globular structure of a double-stranded antiparallel β-sheet with helical turns stabilized by its zinc-binding cluster [149]. It was also noted that residues R227-V230 of the NS1^C-term^ form a third antiparallel β-sheet that is paired with MORC3-CW’s. This third antiparallel sheet includes characteristic β-sheet hydrogen bonds between R227 and K229 of NS1 and W410 and Q412 of MORC3-CW. K229 of NS1^C-term^ is caged between W410 and W419 of MORC3-CW while A226 of NS1^C-term^ settles in a well-defined pocket of MORC3-CW. A comparison of the MORC3-CW:NS1^C-term^ and MORC3-CW:H3K4me3 complexes yields an RMSD of 0.2 Å, indicating that the NS1^C-term^ is an authentic mimic of H3K4me3.

MORC3-CW interacted with the NS1^C-term^ (residues 222-230) with a K_D_ of 157µM, while using NS1^K229me3^ as the target ligand increased the binding affinity (K_D_ = 14 µM) [149]. This increased binding affinity is comparable to the binding affinity of MORC3-CW to histone H3 and the ATPase domain (7 µM and 9 µM, respectively). However, it is still weaker when compared to MORC3-CW’s binding affinity to H3K4me3 (0.6µM). This suggests a mechanism whereby NS1 outcompetes the release of MORC3’s autoinhibition by preventing the interaction between MORC3-CW and the adjacent catalytic domain. Mutating W410 or W419 of MORC3-CW, the residues involved in caging K229 of the NS1^C-term^, to alanine abrogated its binding to the NS1^C-term^. Furthermore, mutating E431 of MORC3-CW to alanine diminished its binding to NS1^C-term^ but had no effect on its interaction with H3K4me3. E431 was selected for study due to its projected proximity to residue 224 of the NS1^C-term^, which was not included in the chimera.

MORC3 has previously been shown to interact with two of the influenza RNA-dependent RNA polymerase (RdRP) subunits (PB1 and PA) as well as with viral RNPs [150,151]. Different studies have shown both positive and negative effects of MORC3 on the virus that were studied through the lens of this polymerase association. Ver et al. demonstrated that MORC3 silencing with shRNA resulted in a significant reduction in viral transcription and viral titers [151]. On the other hand, Bortz et al’s knockdown of MORC3 with siRNA found that viral polymerase activity was significantly increased with a mild increase in viral titers [152]. Zhang et al showed that overexpression of MORC3 resulted in a slight increase in virus infectivity that depended on functional MORC3-CW and ATPase domains, supporting the former study [149]. This dependence is thought to be due to its interaction with the NS1^C-term^. However, further studies will be necessary to fully understand the effect of MORC3 on the viral lifecycle.

## 11. CPSF30

One of NS1’s most studied interactions is that with the host cleavage and polyadenylation specificity factor 30kDa subunit (CPSF30 or CPSF4). CPSF30 is found in the nucleus, where it is involved in mRNA processing and maturation. CPSF, as its name suggests, is involved in both cleavage and polyadenylation of pre-mRNAs through binding the RNA, recruiting other host factors, and catalyzing the cleavage step [153]. NS1 binding to the second and third zinc finger (F2F3) of CPSF30 prevents these functions and results in a global downregulation of host genes. These include interferon and interferon-stimulated genes that ultimately contribute to NS1’s function as a virulence factor for influenza infection [41]. Importantly, this downregulation does not affect viral genes, as their mRNAs are polyadenylated through stuttering of the viral polymerase [154]. Abolishing the interaction between NS1 and F2F3 results in a 10-fold decrease in viral replication and an increase in IFNβ mRNA production [68]. This result alone highlights this interaction as a potential target for novel antiviral development, a process which is aided by the presence of a high-resolution complex structure.

**Figure 7:**
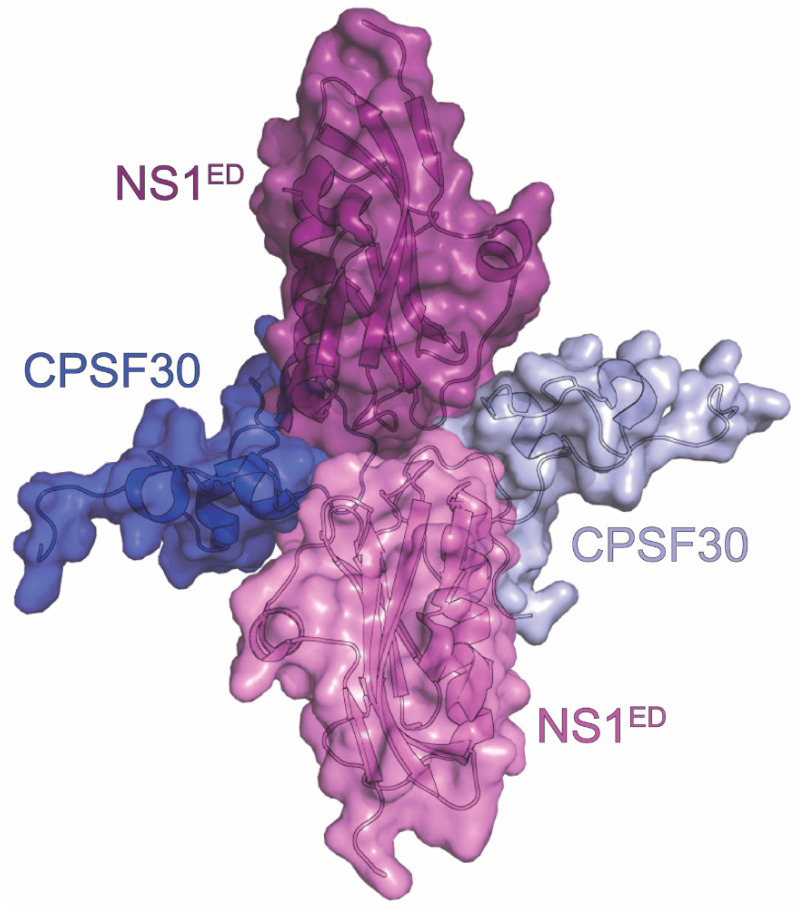
Crystal structure of CPSF30 in complex with NS1. The tetrameric complex is composed of two NS1^ED^s derived from the A/Udorn/307/1972 (H3N2) strain of influenza (violet and light violet) and two F2F3 domains of CPSF30 (blue and light blue). The PDB ID for this structure is 2RHK.

To this end, a crystal structure was solved for F2F3 of CPSF30 bound to the NS1^ED^ (residues 85-215) at 1.95 Å resolution [40]. The overall structure is a tetramer, with two F2F3 molecules wrapped around two NS1^ED^s (Figure 7). The NS1^ED^s in the structure interact with each other in a head-to-head orientation which differs from dimer interfaces previously observed for the NS1^ED^. The F2F3 binding pocket of the NS1^ED^ is largely hydrophobic and very well-conserved among human IAVs. The NS1^ED^ residues critical to the interaction, according to the structure, are K110, I117, I119, Q121, V180, G183, G184, and W187. These residues interact with the aromatic side chains of Y97, F98, and F102 of the corresponding F2F3 protein. Additionally, F103 and M106 were found to be important for stabilization of the complex despite them being outside of the binding pocket. Specifically, the side chain of M106 is positioned in the middle of the tetramer and interacts with the M106 of the other NS1 and residues in both F2F3 molecules. F103’s aromatic side chain of the NS1^ED^ interacts with the hydrophobic residues L72, Y88, and P111 from the adjacent F2F3 molecule. The structure highlights several important regions and residues that could be targeted to hinder NS1’s interaction with CPSF30.

There have been many in vitro and in vivo studies that have identified residues in NS1 that modulate this interaction in several different strains and hosts. In the seasonal H3N2 strain, the I64T mutation in the NS1^RBD^ was found to decrease binding to CPSF30 and increase interferon levels. As the NS1^RBD^ was not included in the crystal structure, the structural mechanism of how this mutation affects the interaction between the NS1^ED^ and F2F3 remains unclear [155]. M106 and F103 have also been shown in numerous studies to be critical to the interaction, but the effects of mutation vary depending on strain and host species. For example, the L103F and I106M mutations in the H7N9 strain restored CPSF30 binding and enhanced virulence. For the 2009 pandemic H1N1 strain, however, restoring the interaction between the NS1^ED^ and CPSF30 impaired virulence [62,156,157,158,159,160].

Loss of binding is commonly seen in avian-to-human transmission, such as with H5N1 and H9N2, suggesting a role in adaptation to mammalian hosts. The H5N1 strain HN01, which lacks CPSF30 binding, is able to recover binding and subsequently decrease interferon activity in human cells with the mutations K55E, K66E, and C133F [161]. Similarly, the HK/97 H9N2 gained binding to CPSF30 and inhibition of host gene expression with the mutations L103F, I106M, P114S, G125D, and N139D [162]. In the swine H5N1 SW/FJ/03, a naturally occurring deletion from 191 to 195 decreased binding to CPSF30 and attenuated virulence in chickens [163]. Some of the strains that lack binding to CPSF30 have evolved to compensate in different ways. The H5N1 strain HK97 lacks binding due to mutations in the stabilizing residues F103 and M106, while descendant strains have lost this defect. HK97 is still able to infect, however, due to compensatory stabilization by its viral polymerase [158,160]. The mouse-adapted H1N1 strain PR8 (PR8^H1N1^) has evolved a different mechanism to make up for a hydrophilic residue at position 103. Since it cannot globally downregulate host genes, it suppresses IRF-3 activation to suppress production of IFN-β mRNA at an earlier step [159]. This has been selected against during replication in humans, suggesting that CPSF30 binding is important for circulating human strains. Collectively, these results highlight the critical role of NS1’s interaction with CPSF30, along with sites that could be targeted by novel antivirals to decrease virulence.

## 12. NXF1-NXT1

NXF1-NXT1 (also known as TAP-p15) is a heterodimeric mRNA export receptor that binds to both mRNA and nucleoporins. This function allows it to shuttle mRNAs through the dense phenylalanine-glycine (FG) repeats of the nuclear pore complex (NPC) into the cytoplasm [164,165,166]. The retinoic acid early inducible 1 protein (Rae1) also participates in the complex and is thought to mediate NXF1’s interaction with mRNAs [167,168,169]. Rae1 facilitates the interaction between NXF1 and mRNAs by recruiting both the nuclear pore complex protein Nup98 and E1B-AP5 into the complex. Both Rae1 and Nup98 are induced by interferons upon viral infection, increasing nuclear export of mRNAs of interferon-stimulated genes such as IFIT2, IRF-1, MHC1, and ICAM-1 [169,170,171,172]. This enhancement makes this pathway a popular target of viral proteins evolved to inhibit the induction of the immune system [171,173,174]. While the influenza A virus already targets mRNA export through the processing machinery (CPSF30), it has also developed a two-pronged approach via interaction with NXF1-NXT1 of the nuclear export machinery.

**Figure 8:**
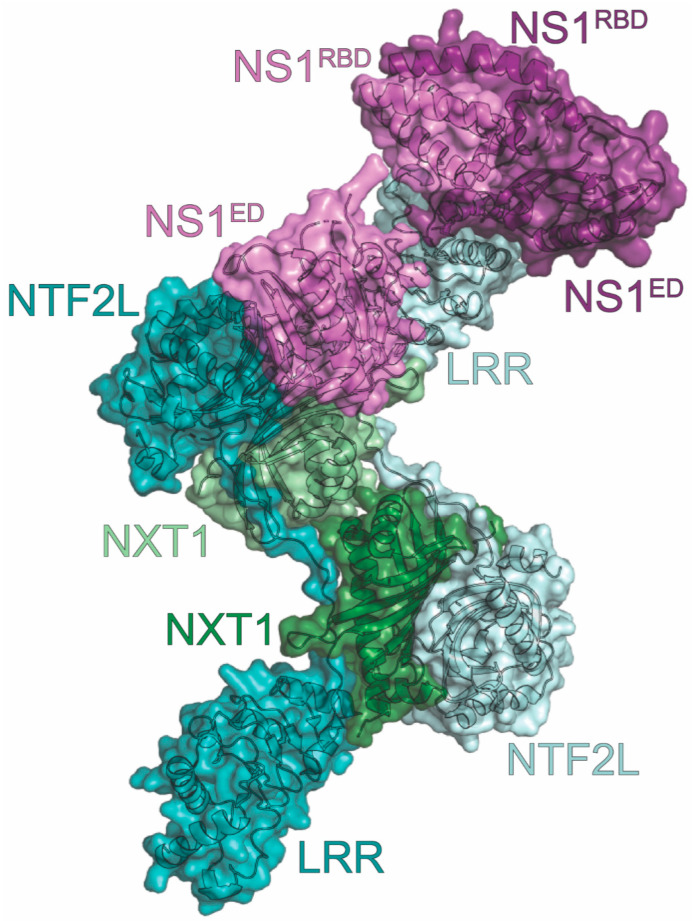
Crystal structure of the NXF1:NXT1 heterodimer in complex with NS1. The complex consists of two copies each of NS1 (violet and light violet), NXF1 (teal and light teal), and NXT1 (green and light green). The NS1 protein is derived from the A/Texas/36/1991 (H1N1) strain of influenza. The nucleoporin-binding domain (NTF2L) and leucine-rich repeat domains (LRR) of NXF1 are labeled. The PDB ID for this structure is 6E5U.

The crystal structure of NS1 complexed with NXF1-NXT1 (resolution at 3.8 Å) revealed a 2:2:2 orientation (Figure 8) [172]. To avoid aggregation and structural polymorphism of NS1, the linker between the NS1^RBD^ and NS1^ED^ was shortened, and two mutations were engineered into NS1 (R38A/K41A). The NXF1 construct used for crystallization only included the leucine-rich repeat (LRR) and nuclear transport factor 2-like (NTF2L) domains. The tetramer, consisting of two NXT1s, each interacting with an LRR and NTF2L from each NXF1, is capped on one end by NS1 dimerized through its NS1^RBD^s. One NS1^ED^ interacts with NTF2L from one NXF1 (interface I), while the other NS1^ED^ interacts with the LRR from the other NXF1 (interface II). The other end of the NXF1-NXT1 tetramer is unoccupied, potentially leaving room for another NS1 dimer. Two highly conserved NS1 residues were identified to be critical for complex formation. F103, located in the α1-β2 loop of the NS1^ED^, inserts into a hydrophobic pocket on NXF1-NTF2L consisting of L383, L386, L491, P521, and L527 to form interface I. F138, located in the β4-β5 loop of the NS1^ED^, binds NXF1-LRR residues K213, M216, Y220, N263, and I264 to form interface II. Additionally, NS1 was found in the same binding site as a nucleoporin FG peptide. It was confirmed through binding analyses that NS1 displaces Nup98 binding to the complex [172,175].

Both IAV infection and ectopic expression of NS1 inhibit the nuclear export of mRNA. This inhibition can be blocked with expression of NXF1, NXT1, Rae1, or Nup98, as well as engineering specific mutations into NS1 (F103A or F138A) [169,172]. These mutations result in a greatly attenuated virus with reduced viral protein levels and replication. Conversely, infections in cells with low levels of Rae1 or Nup98 are more virulent and cytotoxic [169]. The mRNA export pathway has an important role in regulating innate and adaptive immunity, which IAV NS1 blocks to replicate more efficiently. The crystal structure reveals that NS1 enhances IAV replication efficiency by outcompeting Nup98 binding to the complex, thereby blocking nucleoporin binding. These data identify two residues and NS1 regions that could be targeted in the development of anti-influenza therapeutics.

## 13. PDlim2

NS1 proteins derived from some strains of IAV encode a PDZ binding motif (PBM) at their extreme C-terminus. This region is responsible for interactions with many different PDZ-domain containing proteins within the cell, including Scribble, which is discussed above. The sequence of this PBM differs depending on the origin of the strain. For example, most NS1 proteins derived from avian strains, including the highly pathogenic H5N1 strain, encode the PBM ESEV. In contrast, most human strains encode the PBM RSKV or RSEV at this position [141]. This leads to strain-dependent differences in their target proteins that could be responsible for observed changes in pathogenicity upon swapping these sequences. One such interaction is with PDlim2 (aka Mystique or SLIM), a PDZ scaffolding protein involved in the regulation of the cytoskeleton and transcription factors. PDlim2 was found to bind specifically to NS1 derived from the avian H5N1 strain but not to the NS1 derived from an H1N1 strain [176,177]. Structural studies were therefore performed to determine if this difference in binding explains the differences in pathogenesis observed between these two strains.

**Figure 9:**
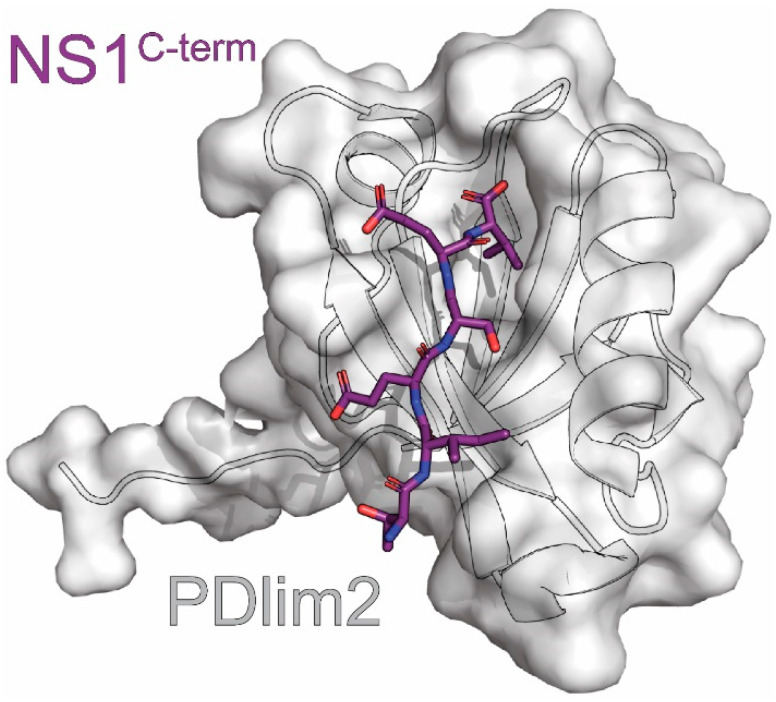
Crystal structure of PDlim2 in complex with NS1. The complex consists of the PBM of NS1 derived from the A/Chicken/Henan/12/2004 (H5N1) strain of influenza (purple) and the PDZ domain of PDlim2 (grey). The PDB ID for this structure is 3PDV.

Yu et al. solved a crystal structure of the PDZ domain of PDlim2 fused to the C-terminal hexapeptide of the NS1^C-term^ derived from the avian H5N1 strain at 2.2Å resolution. (Figure 9) [176,178]. They observed an archetypical PDZ domain with five β-strands and three α-helices, with the helices capping the open ends of the β-sheet barrel. The interaction site was occupied by the NS1^C-term^ hexapeptide from the neighboring fusion protein in the crystal lattice. Residues 0 to -5 of the NS1^C-term^ hexapeptide formed a β-strand that paired with PDlim2 β2 in an anti-parallel manner. A more granular analysis of the structure showed the following: 1) the NS1 residue in the -2 position formed a hydrogen bond with H62 from the α3 helix of PDlim2, 2) glutamines encoded by NS1 in the -1 and -3 positions were stabilized by salt bridges involving R16 and K31 of PDlim2, and 3) the valine encoded by NS1 in the 0 position was nestled in a hydrophobic pocket of PDlim2 consisting of F15 and I17 from the β2 strand, I69 from the α3 helix, and residue W13. These interactions are consistent with previous PDZ-ligand structures from both fusions and co-crystallizations [178,179].

The interactions between NS1 residues in the -1 and -3 positions and PDlim2 residues Arg16 and Lys31 have proven to be particularly important. The -3 position determines differences in PDlim2 binding between strains HN12 (E-3) and PR8^H1N1^ (R-3). Mutating only this position to the other strain’s residue reversed binding between the two both in vitro and in vivo [176]. Similarly, mutating the PDlim2 sites in this interaction greatly affected binding. Both R16A and K31S showed reduced binding, while a double mutant completely abolished it [176]. R16Q was unable to bind even in the presence of K31, indicating a steric requirement. The importance of the salt bridge to this interaction, as shown with mutational analysis and natural variation between strains, makes it a promising candidate for inhibiting NS1 function.

Although we now have knowledge of how to interrupt this interaction, its biological significance is still not understood. To date, two separate PDlim2 functions have been interrogated to no effect. Although PDlim2 functions as an E3 ligase for the p65 subunit of NF-κB and NS1 is known to prevent NF-κB activation, a reporter assay showed no difference between HN12-NS1 WT or ∆PBM in the absence or presence of PDlim2 [176,180,181]. Similarly, HN12-NS1 WT and ∆PBM both resulted in similar levels of STAT1 phosphorylation despite PDlim2’s role in phosphorylation and degradation of STAT1 and STAT4 [176,182]. It is possible that the latter’s lack of effect could be due to the different strain used compared to previous studies and differences between infection and transfection. Additional research is needed to investigate the interaction’s effects on PDlim2’s other functions, such as cell migration or its other roles in the immune system, including through the STAT2 and STAT3 pathways [183,184,185]. Further research is needed to determine the importance of the PDlim2-NS1 interaction, but the information gathered so far will serve as useful tools in this endeavor.

## 14. PI3K

Phosphatidylinositol-3-kinase (PI3K) is targeted by many viruses due to its pivotal role in host survival through various processes, such as apoptosis regulation, metabolism, and immunity [186,187]. When activated, its catalytic subunit p110 converts PIP2 to PIP3, which acts as a secondary messenger to stimulate various pathways such as Akt [188,189]. During homeostasis, p110 is inhibited by PI3K’s regulatory subunit p85 [189,190]. When adaptor proteins bind to the SH2 domain of p85, PI3K is recruited to the plasma membrane where p110 initiates catalysis of PIP2. NS1 has been shown to activate PI3K by mimicking this process and binding to the inter-SH2 domain of p85 [50,188,191,192]. It is this activation of PI3K by NS1 that enhances virulence through delaying apoptosis and/or changing the distribution of PI3K [20,50,191]. However, Jackson et al. posits that NS1’s apoptotic effects are not due to its interaction with PI3K [51]. Additionally, one study linked the interaction to changes in the conductance of lung epithelia [193]. Many studies have been performed to address how this interaction occurs at the residue level and how it contributes to viral pathogenesis.

**Figure 10:**
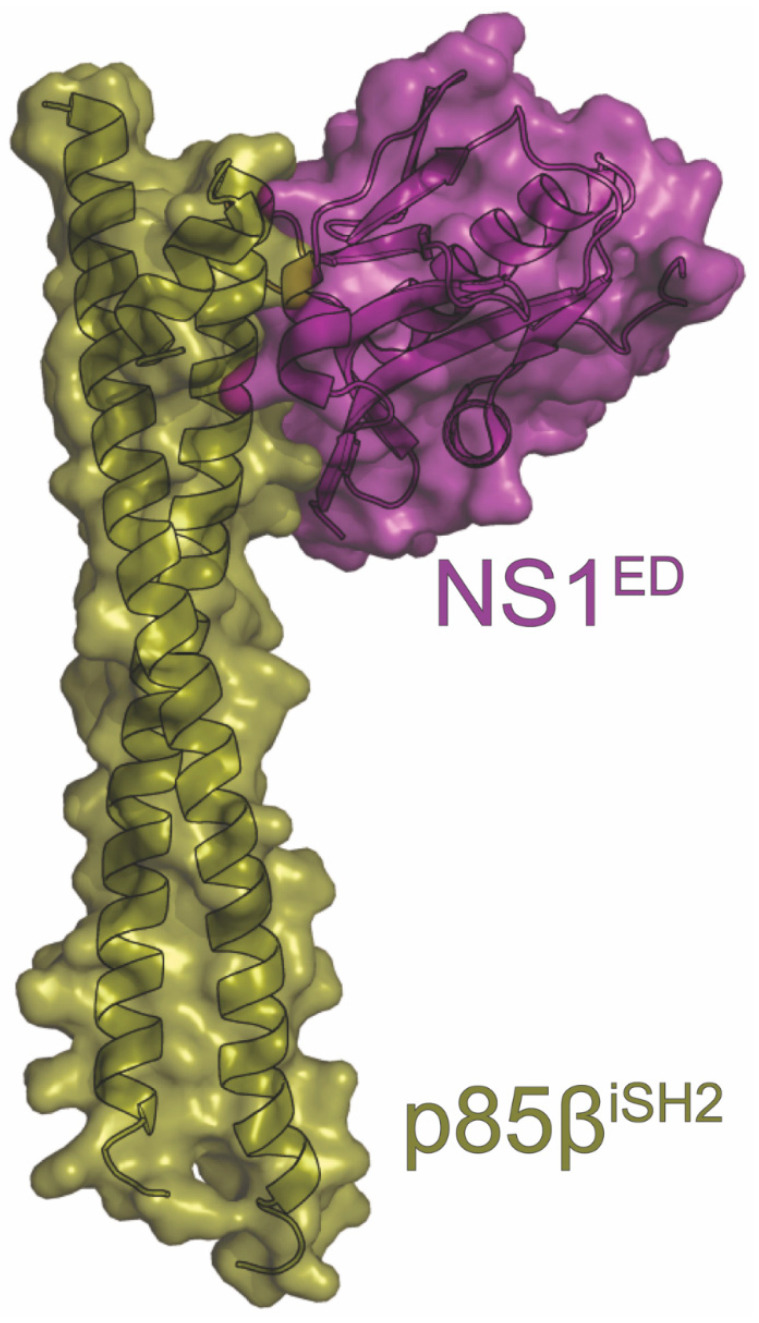
Crystal structure of PI3K in complex with NS1. The complex consists of the NS1^ED^ derived from the A/Brevig Mission/1/1918 (H1N1) strain of influenza (purple) and the p85b^iSH2^ subunit of PI3K (gold). The PDB ID for this structure is 6U28.

The NS1^ED^ binds to the β isoform of PI3K’s p85 regulatory subunit (p85β) [188,191]. This interaction has been structurally analyzed via X-ray crystallography, revealing many details that will provide insight for further studies (Figure 10). To date, multiple structures have been solved using NS1^ED^s derived from multiple strains of IAV and the inter SH2 (iSH2) domain of p85β. Specifically, Hale et al. used the NS1^ED^ derived from the PR8 strain of influenza (PR8^H1N1^ NS1^ED^) and bovine p85β, while Cho et al. used the NS1^ED^s derived from the 1918^H1N1^and Udorn strains of influenza (1918^H1N1^ NS1^ED^ and Udorn^H3N2^ NS1^ED^, respectively) and human p85β [49,194]. They revealed a “golf club-shaped” complex formed from the largely hydrophobic interaction between the NS1^ED^ and one end of the iSH2 coiled-coil, notably at the opposite end from p110’s binding site. Both groups’ structures illustrated the importance of the conserved NS1 residue Y89, as it formed a crucial hydrogen bond with D575 of iSH2 at the center of the complex [49,194]. Previous studies have shown that Y89 is necessary for binding. Specifically, the Y89F mutation in NS1 is unable to pulldown p85β, reduces phosphorylated Akt, and has small plaques and low titers compared to wild-type NS1 [51,188,191,195]. The structure also explained why NS1 has evolved to bind p85β over the more ubiquitous p85α [49,196]. In the complex structure, p85β Val579 lies at the interface, burying 70Å^2^ upon complex formation [49]. In p85α, position 579 encodes a methionine, which sterically hinders the interaction. Superimposing the NS1^ED^:p85β structure onto that of p85α:p110 reveals that the NS1^ED^ sterically prevents p85 SH2 inhibition of p110, explaining the mechanics behind IAV-induced PI3K activation [49]. These structures support and elaborate upon previous work in the field, giving a mechanistic explanation of the interaction that could be used to target this NS1 function for future development of IAV antivirals.

Further analysis of the binding characteristics of the 1918^H1N1^ NS1^ED^ and Udorn^H3N2^ NS1^ED^ with p85β suggests that differences in binding kinetics may contribute to the observed differences in the pathogenicity of each strain [194,197]. In solution, the 1918^H1N1^ NS1^ED^ transiently samples both a binding competent state and binding incompetent state on a sub-millisecond timescale [194]. However, the Udorn^H3N2^ NS1^ED^ is much less dynamic, with its native conformation resembling the binding competent state [194]. Although the K_D_ of the Udorn^H3N2^ NS1^ED^ is six times lower relative to the 1918^H1N1^ NS1^ED^, the 1918^H1N1^ NS1^ED^ hijacks PI3K much more efficiently due to differences in their k_on_ and k_off_ rates [194]. The rapidity with which the 1918^H1N1^ hijacks PI3K could be important for outrunning the innate immune system, contributing to its increased pathogenicity.

Although structural studies with purified domains have garnered important information about complex formation, this interaction is more complicated in the context of the cellular environment. Since p110 is tethered to p85, p110 could be present in the NS1:p85 interaction. A heterotrimer was able to form in vitro and in vivo that proved active during a PI3K assay [192,196]. This is compatible with the X-ray crystal structure, as NS1 and p110 bind at opposite ends of p85. Additionally, NS1 proteins from some strains of influenza can form a heterotrimer with p85β and Crk proteins, discussed previously, in a manner that further enhances PI3K activation [133,198]. SH3 binding competent NS1 proteins were able to displace endogenous p85β-CrkL complexes and form a heterotrimer with NS1 as the bridge. NS1 proteins that could not bind Crk were still observed forming a trimer with p85 as the bridge when p85 was overexpressed [133,198].

Whether via apoptosis inhibition or some other mechanism, NS1-induced PI3K activation is critical for influenza virus replication. This is unsurprising given the vast number of processes PI3K is involved in [188,191]. Further work needs to be undertaken to settle the debate of which PI3K process’s disruption proves most advantageous for the virus. The complex structures should be helpful in this endeavor and will serve as jumping off points for other regions of interest that could be targeted to inhibit NS1.

## 15. RIG-I

NS1’s most well-known function is antagonism of the retinoic acid-inducible gene I (RIG-I) pathway to inhibit the innate immune response. RIG-I is the main cytoplasmic sensor of RNA virus infections, and, as such, it is a crucial part of the immune system’s response to IAV [43,199]. RIG-I consists of two N-terminal caspase activation and recruitment domains (CARDs), a central helicase domain, and a regulatory C-terminal domain. In a normal cellular state, the CARDs are bound to the regulatory domain [200]. Upon binding to 5′-triphosphorylated RNAs, as would be present during IAV infection, a conformational change occurs that exposes the CARDs to ubiquitination by TRIM25 [46,200,201,202]. RIG-I then translocates to the mitochondria where a CARD-CARD interaction occurs with MAVS, leading to the recruitment of kinases IKK and TBK1 to phosphorylate the transcription factor IRF3, which finally moves to the nucleus to promote transcription of type I interferons [203,204,205]. Since RIG-I is the initiator of this long pathway, many viruses have evolved methods to target it to prevent activation of the innate immune response [206,207]. IAV antagonizes the interferon pathway through direct binding of NS1 to RIG-I [44,62,208].

An interaction between purified CARD2 of RIG-I and NS1^RBD^ was directly observed using NMR chemical shift perturbation (CSP) [95]. Interestingly, the binding interface was determined to be localized to the α1 and α3 helices, a functionally novel site on the NS1^RBD^ opposite from the RNA binding interface [95]. Additionally, the interaction was determined to be strain specific when it was observed that perturbations were only observed when using the NS1^RBD^ derived from the 1918^H1N1^ strain but not from the Udorn^H3N2^ strain. Comparing the solution structures of the two RBDs attributed this to a change in orientation of the α3 helix. Specifically, this change in orientation was due to a salt bridge present in the 1918^H1N1^ NS1^RBD^ between residues E72 and R21 that was absent in the Udorn^H3N2^ NS1^RBD^. Mutating the Arg at position 21 to a Glu (as encoded by the Udorn^H3N2^ strain) led to the abrogation of the interaction between the NS1^RBD^ and CARD2 of RIG-I [95]. Overall, the structural confirmation of NS1-RIG-I binding was an exciting finding that was made even more significant by the discovery of a novel binding interface.

Several NS1 residues have been observed to be important for their interactions with RIG-I. Firstly, the aforementioned salt bridge between R21′ and E72 was seen in the solution structure to be crucial for proper placement of the 1918^H1N1^NS1^RBD^ α3 helix to facilitate binding to CARD2. Mutational analysis of the 1918^H1N1^and Udorn^H3N2^ NS1^RBD^ demonstrated that R21 is necessary, but not sufficient, for CARD2 binding, as 1918^H1N1^ R21Q binding was greatly inhibited but not completely removed while Udorn^H3N2^ Q21R was still unable to interact [109]. Viral studies in A549 and HEK293T cells revealed that R21Q exhibited reduced interferon antagonism due to a lessened inhibition of CARD ubiquitination by TRIM25 [109]. Secondly, two NS1 mutations important for host adaptation and virulence in the H5N1 strain HK97 were shown to affect RIG-I binding [62]. F103L and M106I were investigated in the WSN background using a bacterial reverse two-hybrid system. While F103 and M106 could not interact with any of the RIG-I subunits, different combinations of the two mutations restored binding to up to all three subunits: the double mutant bound to the regulatory domain and CARD, F103L bound CARD and helicase, and M106I bound all three [62]. All three of the above mutations are promising avenues to explore to better understand the NS1-RIG-I interaction.

NS1’s crucial function of RIG-I antagonism allows for IAV to gain a foothold in cells through interrupting not only the innate immune response but also the inflammasome, through RIG-I’s binding to ASC and caspase 1 [209]. This is at least partly achieved through preventing ubiquitination of the CARDs by TRIM25, perhaps by physically blocking access to the CARDS by TRIM25 and/or free-floating ubiquitin chains [95,109]. However, there have also been reports of direct NS1 binding to the other RIG-I subunits, the regulatory domain and helicase, along with a potential complex of NS1, RIG-I, and MAVS, implying that there is more to this mechanism that is yet to be discovered [44,62,208]. Whatever the mechanism may be, the importance of the interaction for IAV virulence has been proven numerous times. Transfection of RIG-I into infected cells significantly decreases viral titers, while ∆NS1 infections produce drastically more interferon and are cleared much faster, an effect which is reversible through RIG-I silencing [43,45,46]. Given its importance, more structural studies should be performed to determine the extent to which the other subunits are involved in order to obtain the full picture of this mechanism. The NS1-RIG-I interaction is an enticing antiviral target, but there are still gaps in our knowledge that could be further illuminated.

## 16. TRIM25

As discussed above, NS1’s ability to antagonize the innate immune response through inhibition of the RIG-I pathway is among its most important contributions to IAV virulence. It therefore stands to reason that to completely suppress this pathway, multiple points would be targeted. One other target in this system is tripartite motif-containing protein 25 (TRIM25). The TRIM family of proteins are RING-type E3 ligases that are involved in pathways leading to type-I interferon and inflammatory cytokine production. In fact, TRIM25 is specifically required to activate the RIG-I pathway. When the cytosolic sensor RIG-I binds to viral RNA, a conformational change exposes the CARD domains, allowing TRIM25 to bind to CARD1 and ubiquitinate K172 in CARD2 [201,202]. The ubiquitinated CARDs allow for interaction with MAVS, triggering the downstream effects of the pathway [203,204]. TRIM25 consists of a catalytic RING domain, one or two B-box domains, and a coiled-coil (CC) domain [210]. A C-terminal PRYSPRY motif mediates the interaction with CARD1 [202,211]. Co-IP, confocal microscopy, and bacterial real-time hybrid simulation (RTHS) experiments identified that IAV NS1 directly binds to TRIM25 to antagonize interferon production. However, there was some debate over how this binding occurred, along with its precise effects, which structural studies sought to address [208,212,213,214].

**Figure 11:**
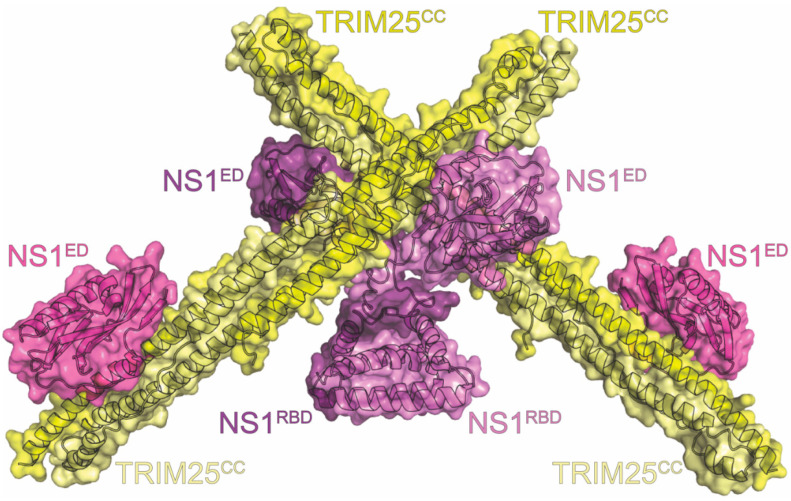
Crystal structure of TRIM25 in complex with NS1. The complex consists of the NS1 homodimer (light violet and violet), two copies of the coiled-coil region of TRIM25 (TRIM25^CC^) colored light yellow and yellow, and two additional copies of the NS1^ED^ (mauve). The NS1 protein in the structure is derived from the A/Puerto Rico/8/1934 (H1N1) strain of influenza. The PDB ID for this structure is 5NT1.

Crystal structures of purified TRIM25-CC complexed with both full-length and the NS1^ED^ were obtained at 4.26Å and 2.8Å resolution, respectively. We note that the NS1^ED^ alone was sufficient for binding and that each purified NS1^ED^ bound to a TRIM25-CC dimer at two separate interfaces [114]. However, the second interface was not present with full-length NS1(Figure 11). This was additionally confirmed via mutational analysis [114]. The remaining interface consists of a linker between TRIM25-CC helices α2 and α3 making contacts with a highly conserved NS1 motif that includes a short α-helix comprising residues 95-99. NS1 L95 was especially important in the interaction, as it contacted a hydrophobic pocket of TRIM25 formed by residues from both chains of the CC, including V223, F274, I277, I324, and V327. L95 also participates in a hydrogen bond with TRIM25-CC E326. Other interactions can be observed between Y89_ED_, D222_CC_, and D229_CC_, as well as E101_ED_ and Q212_CC_ [114].

The complex structures cleared up some misconceptions about the mechanics of this interaction. While it was previously thought that NS1 binding to the TRIM25-CC would prevent TRIM25 dimerization, which is needed for activity, the complex showed that the dimer itself was involved in the interaction [114,212]. The dimeric TRIM25-CC is bound by two NS1^ED^s symmetrically at each end. This arrangement, along with NS1 being an obligate homodimer, was hypothesized to mediate higher order oligomer chains of NS1 and TRIM25, leading to more efficient sequestration [114]. This hypothesis is supported by the finding that mutating crucial NS1 residues L95, S99, and Y89 identified in the structure severely impacted the interaction while only slightly affecting RIG-I ubiquitination and interferon production. It was posited that even lower than expected binding affinity for each individual interaction can be overcome by oligomerization [114]. Previously investigated NS1 residues were not seen to be involved in the binding interface. E96/E97, which, when mutated, lost TRIM25 interference and virulence in mice, actually faced away from the interface and are likely needed for NS1 structural integrity rather than binding [114,212]. R38/K41 are known to be involved in RNA binding and RBD dimerization, meaning that, in this context, they likely interfere with higher-order oligomerization rather than direct TRIM25 binding as previously suggested [114,212].

Superposition of the structures with that of TRIM25CC-PRYSPRY revealed a different mechanism for NS1’s antagonism. Both PRYSPRY and NS1 ED contacted the linker region between α2 and α3, leading to steric hindrance in the presence of both [114]. Therefore, NS1 is likely to inhibit TRIM25 by preventing PRYSPRY and RING from being in the correct orientation to allow ubiquitination. The interaction was found to be host-species-specific, as NS1s from various strains have adapted to interact more efficiently with their host’s TRIM25 homologue. This adaptation demonstrates how NS1 can help drive host adaptation [213]. By adding another protein into its long list of interactions, NS1 achieves greater coverage of the RIG-I pathway to further antagonize the innate immune system and interferon production.

## 17. Conclusions

The ever-evolving nature of the Influenza A virus has rendered many of our defenses ineffective, making the need for new, innovative antivirals that much more pressing. Taking a structural approach to the development of these new antivirals arms us with the knowledge of how exactly we are targeting the virus, allowing us to make the most informed decisions possible. By knowing the structural underpinnings of crucial complexes for IAV pathogenesis, we can design drugs that target the most important interfaces to have the greatest effects. If we combine that knowledge with genetic data, we can narrow our targets to the most conserved regions of the viral protein that are the least likely to mutate. This process starts with the work covered in this review, but there is still much to be done, as has been highlighted. Some of the complexes discussed are still not fully mapped out, while many other complexes remain structurally unstudied (Table 1). Every piece of data drives us closer to being able to make the most effective IAV antivirals possible, making this field of study important and worthwhile.

## Figures and Tables

**Table 1:** Verified interactions between cellular host proteins and NS1 with the techniques used listed. Abbreviations used in the table are as follows: X-ray = X-ray crystallography, NMR = Nuclear magnetic resonance. Y2H = Yeast two hybrid, SEC-MALS = Size exclusion chromatography with multi-angle light scattering, 2DE = 2D gel electrophoresis, MS = Mass spectrometry, MD = Molecular dynamics simulations, FRET = Fluorescence resonance energy transfer, FA = Fluorescence anisotropy, BLI = Bio-layer interferometry, M2H = Mammalian two hybrid, CD = Circular dichroism, and IP = Immunoprecipitation. Certain techniques listed (e.g., IP) can be performed with either whole cell lysate or purified protein. For these types of experiments, an asterisk (*) denotes that these experiments were performed with purified proteins. Interactions that are defined with single amino acid resolution are denoted by grey shading of the cells.

Name	Host Protein Function	Results of Interaction	Methods	PDB ID	Figure	Citations
CHD1	chromatin remodeling	downregulation of host genes	IP*, ITC, X-ray	4NW2 4O42	3	Marazzi (2012) [87] Qin (2014) [115]
Ci/Gli1	transcriptional mediator in Hedgehog pathway	modulates Hedgehog signaling	FRET-FLIM			Smelkinson (2017) [215]
CPSF30	mRNA processing	downregulation of host genes	co-IP, Confocal, Pulldown, X-ray, Y2H	2RHK	7	Das (2008) [40] Nemeroff (1998) [41] Dankar (2013) [62] Twu (2006) [68] Kuo (2018) [77] DeDiego (2016) [155] Kuo (2009) [158] Kochs (2007) [159] Twu (2007) [160] Li (2018) [161] Rodriguez (2018) [162] Zhu (2008) [163]
CrkII	cellular signaling through binding to JNK1	inhibits innate immune response	Confocal, NMR Relaxation Dispersion, Pulldown, X-ray	5UL6	4	Shen (2017) [116] Ylösmäki (2016) [126]
CrkL	PI3K signaling	inhibits apoptosis	Binding assays *, co-IP			Hrincius (2010) [125] Heikinnen (2008) [127] Ylösmäki (2015) [133] Miyazaki (2013) [216]
Dlg	tight junction formation	disrupts tight junction formation to enhance viral spread	co-IP, Confocal, Pulldown *			Golebiewski (2011) [88] Thomas (2011) [89] Liu (2010) [137]
eIF4E	translation initiation	stimulates translation of viral mRNA	FA			Cruz (2022) [217]
eIF4G1	translation initiation	enhances viral translation	co-IP, Pulldown *			Burgui (2003) [218] Aragon (2000) [219]
hPAF1C	transcription elongation	downregulation of host genes	IP *			Marazzi (2012) [87]
IFI35	RIG-I regulation through degradation	inhibits type I interferon production to inhibit innate immune system	co-IP, Confocal, Pulldown *			Yang (2021) [220]
MAGI-1	tight junction formation	disables tight junction formation to enhance viral spread	co-IP, Confocal, Pulldown *			Thomas (2011) [89] Liu (2010) [137] Kumar (2012) [221]
MORC3	ATPase with antiviral activity	prevents MORC3 histone binding and autoinhibition	NMR, X-ray	6O5W	6	Zhang (2019) [149]
NS1-BP	inhibits splicing, inhibits some NS1 activities	regulates cell viability through blocking NS1 stimulation of ERK pathway	IP *, Pulldown, Y2H			Miyazaki (2013) [216] Wolff (1998) [222]
Nucleolin and Fibrillarin	rRNA production	represses ribosome production	co-IP *, Confocal, Pulldown *			Yan (2017) [223] Melen (2012) [224] Murayama (2007) [225]
NXF1-NXT1	RNA nuclear export	inhibits host mRNA translation	IP, Pulldown *, X-ray	6E5U	8	Satterly (2007) [169] Zhang (2019) [172]
PABP1	translation initiation	enhances viral translation	co-IP, EMSA, FA, FRET, Pulldown *			Burgui (2003) [218] de Rozieres (2020) [226] Arias-Mireles (2018) [227]
PABPII	mRNA processing	downregulation of host genes	IP *			Chen (1999) [228]
PACT	stimulates RIG-I-induced interferon production	inhibits interferon production	co-IP, Pulldown *, SILAC			Li (2006) [47] Tawaratsumida (2014) [229]
PDlim2	scaffold protein that recruits NF-kB to ubiquitin ligase	unknown	BiFC, Pulldown *, M2H, X-ray	3PDV	9	Yu (2011) [176]
PI3K	apoptosis regulation	inhibits apoptosis	BLI, co-IP, Confocal, ITC, MD, NMR, Pulldown, X-ray	6U28 6OX7	10	Hale (2010) [49] Ehrhardt (2007) [50] Ylösmäki (2015) [133] Hale (2006) [188] Shin (2007) [191] Hale (2008) [192] Cho (2020) [194] Li (2008) [196] Dubrow (2021) [197] Dubrow (2020) [198]
PKR	antiviral activity through translational shutdown	continued viral mRNA translation	co-IP, FRET, Pulldown *, Y2H			Li (2006) [47] Min (2007) [48] Schierhorn (2017) [230] Tan (1998) [231]
PSD-95	regulates NO production	reduces NO production	IP *, Pulldown *, Y2H			Zhang (2011) [232]
RIG-I	interferon induction	inhibits interferon production	co-IP, Confocal, Crosslinking IP, NMR, Pulldown, Y2H,			Mibayashi (2007) [44] Dankar (2013) [62] Jureka (2015) [95] Jureka (2020) [109] Pothlichet (2013) [209]
RIL and Src Kinase	kinase cascade for cell signaling	alters cell signaling	Protein arrays, Pulldown *			Bavagnoli (2011) [233]
RuvBL2	ATPase and helicase with multiple regulatory functions	inhibits infection-induced apoptosis by maintaining RuvBL2 protein abundance	2DE-MS, co-IP, Pulldown *			Wang (2021) [79]
Scribble	cell polarity regulator, tight junction formation	disrupts tight junction formation to enhance viral spread	CD, co-IP, Confocal, ITC, Pulldown *, X-ray	7QTO 7QTP 7QTU	5	Golebiewski (2011) [88] Thomas (2011) [89] Liu (2010) [137] Javorsky (2022) [143]
Staufen	RNA translocation and regulation	inhibits viral mRNA degradation	Confocal, IP *, Tethering assay, Y2H			Cho (2013) [234] Lee (2011) [235] Falcon (1999) [236]
TRIM25	RIG-I activation	inhibits innate immune response	BLI, co-IP *, Confocal, NMR, SECMALS, X-ray	5NT1 5NT2	11	Koliopoulos (2018) [114] Gack (2009) [212] Rajsbaum (2012) [213]
WDR5	coactivator involved in gene regulation	downregulation of host genes	ITC, X-ray	4O45	1	Qin (2014) [115]
XRN1	mRNA decay factor in P-bodies	inhibits degradation of viral mRNAs	co-IP, Confocal, Pulldown *			Liu (2021) [237]
YAP/TAZ	Hippo pathway activation	downregulates cytokines and the innate immune system	co-IP with exogenous Protein			Zhang (2022) [238]

## Data Availability

Not applicable.
